# Proteomic profiling of gastric cancer with peritoneal metastasis identifies a protein signature associated with immune microenvironment and patient outcome

**DOI:** 10.1007/s10120-023-01379-0

**Published:** 2023-03-17

**Authors:** Yanyan Chen, Guoxin Cai, Junjie Jiang, Chao He, Yiran Chen, Yongfeng Ding, Jun Lu, Wenyi Zhao, Yan Yang, Yiqin Zhang, Guanghao Wu, Haiyong Wang, Zhan Zhou, Lisong Teng

**Affiliations:** 1 Department of Surgical Oncology, The First Affiliated Hospital, Zhejiang University School of Medicine, 79# Qingchun Road, Hangzhou, 310003 China; 2 Institute of Drug Metabolism and Pharmaceutical Analysis and Zhejiang Provincial Key Laboratory of Anti-Cancer Drug Research, College of Pharmaceutical Sciences, Zhejiang University, Hangzhou, China; 3 Department of Gastroenterology, Affiliated Hangzhou First People’s Hospital, Zhejiang University School of Medicine, Hangzhou, China; 4 Department of Medical Oncology, The First Affiliated Hospital, Zhejiang University School of Medicine, Hangzhou, China; 5 Department of Informatics, The First Affiliated Hospital of Wenzhou Medical University, Wenzhou, China; 6 School of Clinical Medicine, Hangzhou Normal University Medical College, Hangzhou, China

**Keywords:** Gastric cancer, Peritoneal metastasis, Proteomics, Machine learning

## Abstract

**Background:**

Peritoneal metastasis (PM) frequently occurs in patients with gastric cancer (GC) and is a major cause of mortality. Risk stratification for PM can optimize decision making in GC treatment.

**Methods:**

A total of 25 GC patients (13 with synchronous, 6 with metachronous PM and 6 PM-free) were included in this study. Quantitative proteomics by high-depth tandem mass tags labeling and whole-exome sequencing were conducted in primary GC and PM samples. Proteomic signature and prognostic model were established by machine learning algorithms in PM and PM-free GC, then validated in two external cohorts. Tumor-infiltrating immune cells in GC were analyzed by CIBERSORT.

**Results:**

Heterogeneity between paired primary and PM samples was observed at both genomic and proteomic levels. Compared to primary GC, proteome of PM samples was enriched in RNA binding and extracellular exosomes. 641 differently expressed proteins (DEPs) between primary GC of PM group and PM-free group were screened, which were enriched in extracellular exosome and cell adhesion pathways. Subsequently, a ten-protein signature was derived based on DEPs by machine learning. This signature was significantly associated with patient prognosis in internal cohort and two external proteomic datasets of diffuse and mixed type GC. Tumor-infiltrating immune cell analysis showed that the signature was associated with immune microenvironment of GC.

**Conclusions:**

We characterized proteomic features that were informative for PM progression of GC. A protein signature associated with immune microenvironment and patient outcome was derived, and it could guide risk stratification and individualized treatment.

**Supplementary Information:**

The online version contains supplementary material available at 10.1007/s10120-023-01379-0.

## Background

Gastric cancer (GC) is the fifth most commonly diagnosed malignancy and the fourth leading cause of cancer-related mortality worldwide [1]. Peritoneal metastasis (PM) of GC is an aggressive disease with poor prognosis, having limited response to palliative chemotherapy [2]. PM is occasionally found at the time of initial GC diagnosis or during intended radical surgery (synchronous PM). Even after radical surgery, PM occurs as peritoneal recurrence (metachronous PM), accounting for up to 50% of all recurrences [3, 4]. Emerging intraperitoneal treatment could provide survival benefit in locally advanced GC or GC patients with PM [5]. Nevertheless, considering the potential complications of these treatments, more precise evaluation of PM risk in GC patients is crucial to identify those who would benefit from intraperitoneal treatment, optimizing decision making to balance benefits and overtreatment.

Next-generation sequencing enables the high-throughput and systematic analysis of genetic alterations, offering novel insights into molecular basis of oncogenesis and heterogeneity. Importantly, The Cancer Genome Atlas (TCGA) classified GC as four subtypes: Epstein-Barr virus (EBV), microsatellite instability (MSI), chromosomal instability (CIN), and genomically stable (GS) [6]. Our team previously purposed a molecular classification that differentiates GC subtypes associated with prognosis and metastasis patterns towards liver metastasis or PM [7]. However, genomic heterogeneity between primary and metastatic tumor in GC could be extensive [8]. Therefore, to investigate the molecular background of PM propensity and to identify patients with high PM risk, study including primary lesions and corresponding peritoneal metastases remain required.

As proteins are regarded as “executors of life”, proteomic studies provided insight into cancer mechanisms and potential clinical implications. Proteomic profiling of diffuse GC identified three subtypes with different prognosis [9]. Li et al. uncovered proteomic signatures for progression of gastric lesions and risk of early GC [10]. Recent proteomic study of GC undergoing chemotherapy or targeted therapy identified proteomic features to predict therapeutic response [11]. Nevertheless, proteomic characteristics of GC with PM and their clinical relevance have not been extensively studied.

In recent years, cancer immunotherapy using immune checkpoint inhibitors (ICIs) has emerged as promising treatment for specific subgroups of GC [12, 13]. However, the clinical efficacy of ICIs against PM of GC is still unclear. Previous studies have suggested that the response to anti-PD-1 therapy tended to be less prominent in GC with PM [14, 15]. Since the tumor immune microenvironment (TIM) is highly associated with immunotherapy response and prognosis [16], investigation of TIM of GC with PM could potentially reveal relevance to ICI response.

In this study, we sought to investigate the molecular features of GC with PM based on in-depth proteome profiling and whole-exome sequencing of GC. By machine learning, we identified a proteomic signature associated with PM and subsequently generated a prognostic signature, which was validated in external proteomic datasets. This proteomic signature was also associated with TIM and potentially predictive for immunotherapy response. Our findings may have translational significance for guiding decision making in GC management.

## Methods

### Patient samples

A total of 25 GC patients who underwent gastrectomy at the First Affiliated Hospital of Zhejiang University School of Medicine between January 2018 and January 2022 were retrospectively enrolled. 13 patients with synchronous PM underwent palliative surgery for GI bleeding or obstruction. 12 patients underwent curative surgery, among whom 6 developed peritoneal recurrence (metachronous) as the first metastatic site, 6 did not develop PM within 24 months (non-PM group). Patients received standard adjuvant chemotherapy or perioperative systemic treatment (with or without immunotherapy). Follow-up data were obtained by phone and the out-patient clinical database (last follow-up, November 2022). Patient-written consents were obtained from study participants, informing the use for proteomic and genomic profiling and publication. Fresh-frozen, FFPE samples and malignant ascites were collected in accordance with ethical guidelines in the First Affiliated Hospital, Zhejiang University School of Medicine.

### DNA extraction and whole‑exome sequencing

For fresh-frozen samples, QIAamp DNA Mini Kit (Qiagen) was used to isolate genomic DNA from tumor tissues and matched normal mucosa according to the manufacturer’s instructions. For FFPE tumor tissues, DNA was extracted using QIAamp DNA FFPE Tissue Kit (Qiagen). DNA degradation and contamination were monitored on 1% agarose gels. Qubit^®^ DNA Assay Kit in Qubit^®^ 2.0 Flurometer (Invitrogen, USA) was used to quantify DNA concentration. For malignant ascites, tumor content was centrifuged for 20 min at 2000*g* and sediment tumor cells were used for DNA and protein extraction.

Whole-exome library construction was generated using the Agilent SureSelect Human All Exon V6 Kit (Agilent Technologies, Santa Clara, CA, USA). The index-coded samples were clustered on a cBot Cluster Generation System using Hiseq PE Cluster Kit (Illumina). The DNA libraries were sequenced on Illumina Hiseq platform (Illumina, San Diego, California, USA) and 150 bp paired-end reads were generated. We first conducted data quality control and then performed all downstream bioinformatics analyses based on the high-quality clean data. The paired-end clean reads were aligned to the Human Genome Reference Consortium build 37 (GRCh37) using BWA v.0.7.8 [17]. Mapped reads were then de-duplicated using Sambamba tools (v0.4.7) [18].

Identification of somatic single-nucleotide variants (SNVs) was conducted by muTect (v1.1.4), and the somatic InDels were detected by Strelka (v1.0.13). ANNOVAR (ANNOVAR_2015Mar22) was used to annotate variant call format files [19].

### Protein extraction and quantitative proteome profiling

The protein extraction was performed according to Zhang et al. [20]. Samples were minced and lysed in lysis buffer (8 M Urea, 100 mM Tris Hydrochloride, pH 8.0) containing protease and phosphatase Inhibitors (Thermo Scientific) followed by sonication. The lysate was centrifuged and the supernatant was collected. Extracts from sample was reduced with 10 mM dithiothreitol at 56 °C for 60 min and alkylated with 10 mM iodoacetamide at room temperature in the dark for additional 60 min. The samples were digested with trypsin. The digested peptides were desalinated using C18 column (50% acetonitrile and 0.1% formic acid) and resolved in 100 mM triethylamine buffer. Finally, the samples were labeled using the TMT^®^ Mass Tagging Kits and Reagents (Thermo scientific, USA) following the manufacturer’s instructions.

Mass Spectrometry (MS) detection was carried out as previously described [20]. Digested samples were analyzed on Q Exactive HF-X Hybrid Quadrupole-Orbitrap Mass Spectrometer (Thermo Scientific, USA) coupled with a high-performance liquid chromatography system (EASY nLC 1200, Thermo Fisher). MS raw files generated by LC–MS/MS were searched against the NCBI human Refseq protein database using Proteome Discoverer 2.4 (PD2.4, Thermo) software. Then peptide spectrum matches with reliability > 99%, or proteins containing at least one unique peptide, with FDR < 1% were identified.

### Copy number analysis

Somatic copy number variations (SCNVs) were identified using CNVkit [21]. Then GISTIC 2.0 (v 2.0.22) [22] was used to identify the genome regions with significant alterations and screen out the recurrent CNV region.

### Analysis of clonal architecture

The R package SciClone [23] was used to infer the clonal and subclonal architecture of somatic mutations by analyzing the variant allele frequencies in an individual sample using the Bayesian binomial mixture model.

### Identification of differentially expressed proteins

Analysis of differently expressed proteins (DEPs) between tumor and normal was conducted by (a) R package samr (v3.0) with an FDR threshold at 0.05 and a fold change (FC) threshold at 1.5. The results were run under 100 times permutations resampling and visualized by VolcaNoseR [24]; (b) Referring to GFold [25], the DEPs of each sample pair were derived by the following steps. First, for each sample pair, log foldchanges of each gene were fitted to t-distribution. Two-tail test was performed to get DEPs with a *P* value threshold of 0.05. The overlaps of (a) and (b) were regarded as the final DEPs.

### Identification of PM-associated proteins

Our previously developed tool MATTE (v1.2.3) [26] was used to identify the differentiated module between PM samples and without-PM samples based on DEPs. After clustering, all differentially expressed modules were compared by their signal-to-noises (SNRs). The module with the highest SNR was thought as the PM-associated module. External transcriptome data [27] was used for filtering proteins. The classifier for PM samples identification was built by Xgboost (v1.7) [28] and simplified by scikit-learn (v1.2) [29]. Specifically, we first fitted GB-tree classifier by data with previously filtered proteins. By taking the area under the curve (AUC) in fivefold cross validation as the metric, 32 proteins got best score. To further simplify the model, we listed all combinations of proteins and scored them as previously. Each protein’s frequency in the top 500 protein combinations of all was ranked. Ten proteins with the highest frequency in the combinations were selected for the final model.

### Establishment of prognostic model

For previously selected ten proteins, a GB-linear classifier was fitted in our cohort. Then weight of each protein was extracted. The sum of weighted protein expression was calculated as PM risk score. Subjects were stratified into high, moderate and low-risk levels by PM risk score.

### Immune cells infiltration enumerations

Estimation of immune cell types infiltration level was based on sample-level normalization proteome data. Then, the estimation was performed by TIMER web server [30] and the result of CIBERSORT algorithm [31] was used for analysis.

### Statistical analysis

The statistical analysis was performed by SPSS 21.0 software. Two continuous variables were compared using Student’s *t* test. Survival data was analyzed by Kaplan–Meier curves with log-rank test. Univariate and multivariate Cox regression analysis were performed to calculate the hazard ratio and 95% confidence interval. A *P* value of < 0.05 was considered significant.

## Results

### Patient characteristics, proteome profiling and whole exome sequencing

**Fig. 1:**
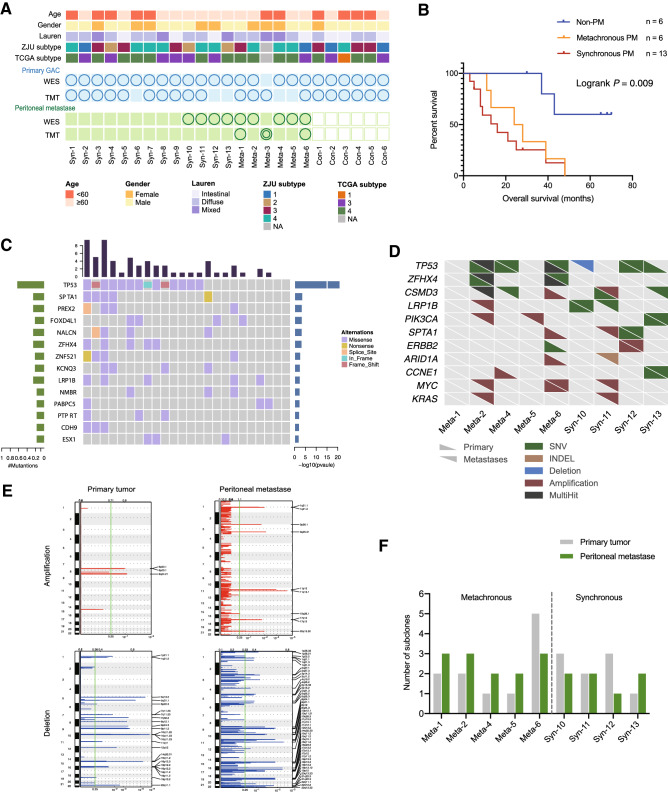
Genomic background of primary GC and paired PM samples. **A** Clinicopathological information and molecular classifications of GC cohort; Circles represent available data. (Dual circles represent two samples collected at different timepoints), **B** Survival analysis of three patient groups, **C** Landscape of somatic mutations of the cohort. Genes with variants in at least 3 patients were depicted. Bars on top and to the right of the graph show the number of non-synonymous mutations in each subject and gene, respectively, **D** Comparison of key somatic mutations and CNV between paired primary GC and PM, **E** Somatic CNV of primary GC and PM samples, **F** Number of subclones of each paired primary GC/PM. *Syn* synchronous metastasis, *Meta* metachronous metastasis, *Con* control group, *WES* whole-exome sequencing, *TMT* tandem mass tags

We enrolled 19 GC patients who developed synchronous (*n* = 13) or metachronous (*n* = 6) PM without other distant metastasis, and 6 GC patients with stage IIIB or IIIC disease who did not develop peritoneal recurrence for at least 24 months after curative surgery (PM-free group). Primary tumor tissue (T), peritoneal metastases (M), and their matching adjacent normal tissues (N) were collected for proteome profiling and/or whole-exome sequencing (Fig. 1A). The overall survival showed significant difference between PM and PM-free groups (Fig. 1B). The detailed information is shown in Table S1. By high-depth tandem mass tags (TMT) labeling for quantitative proteomics, a total of 20 pairs of tumor and normal tissues were measured and the results showed good consistency in proteome identification and quantification (Fig. S1), resulting in the identification of 9852 proteins. To increase reliability, we selected 6799 proteins that were detected with at least 2 unique peptides and normalization to fraction of total (FOT) > 10^−5^. Z-score normalization (log_2_ of relative abundance scaled by subjects’ SD) was performed for comparative analysis.

To obtain genetic background of our cohort, we conducted whole-exome sequencing. The mean coverage of sequencing was 263-fold in tumor and 122-fold in normal samples. A total of 3439 somatic non-synonymous mutations were detected in all sequenced cases. *TP53 (63%), SPTA1 (21%), PREX2 (16%), ZFHX4(21%), LRP1B (21%)* were identified as significantly mutated genes (Fig. 1C). All GC cases were identified as microsatellite stable.

### Genomic and proteomic features in PM in comparison with primary GC

To investigate the heterogeneity between primary GC and PM, we compared multiple genomic features in 9 pairs of samples. All paired samples shared some alterations, confirming that they had the same tumor origin. The proportions of somatic mutations shared with paired PM ranged from 9 to 62% in primary GC (Fig. S2). Mutations of *TP53* and *ZFHX4* amplifications were generally shared between primary tumor and PM, while *CSMD3* mutations, CNVs were frequently discrepant, such as potentially targetable amplifications of *CCNE1, MYC* and *KRAS* (Fig. 1D). Notably, compared with primary tumor, PM samples harbored more recurrent CNV (Fig. 1E). Mutational signature and neoantigen were generally concordant in paired primary and PM samples (Figs. S3–4). The number of subclones was frequently discrepant in PM and primary GC, reflecting different intratumor heterogeneity. Notably, number of subclones in PM increased in 4 out of 5 patients with metachronous PM (Fig. 1F). The above results confirmed high genomic heterogeneity between primary GC and PM samples.

**Fig. 2:**
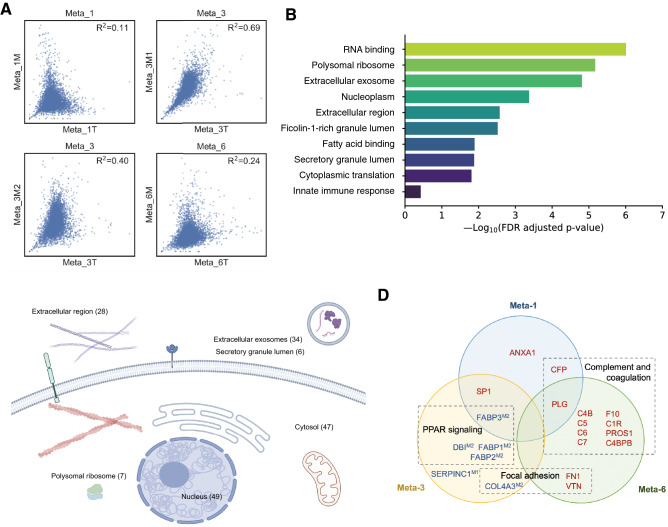
Proteomic features of paired primary GC and PM. **A** Scatterplot showing correlation of protein abundance in paired primary GC/PM samples, **B** Top ranked pathways that are significantly enriched in PM as compared with primary GC, **C** Subcellular distribution of proteins enriched in PM annotated with Gene Ontology, **D** Venn diagram presenting altered proteins (up- or downregulated in both primary and PM samples) in each subject and relevant pathways (for Meta-3M, proteins altered in superscript samples)

Four PM samples derived from malignant ascites were analyzed at proteome level (two samples were collected from Meta-3 at different timepoints). The protein abundance between paired primary GC/PM tissue showed relatively low correlation (Fig. 2A). Compared with primary GC samples, we identified 118 proteins enriched in PM samples (Table S2). A large number of these proteins distributed in extracellular region, including exosome and secretary granule (Fig. 2B). Pathway analysis revealed that these proteins were enriched in RNA binding, polysomal ribosome and extracellular exosome (Fig. 2C). We further focused on proteins that showed the same expression trend in primary and PM samples. PLG, SP1 and FABP3 were consistently up- or downregulated in two pairs of primary GC/PM, and there were multiple altered pathways overlapped across cases, including focal adhesion and complement pathway (Fig. 2D). Specifically, Meta-3M2 (24 months after radical surgery, during second-line treatment) had more altered proteins associated with signaling pathways than Meta-3M1 (10 months after radical surgery, first-line chemotherapy completed), suggesting that more mechanical changes might be induced through tumor progression and additional treatment.

### Characteristic alterations in proteome of GC associated with PM propensity

**Fig. 3:**
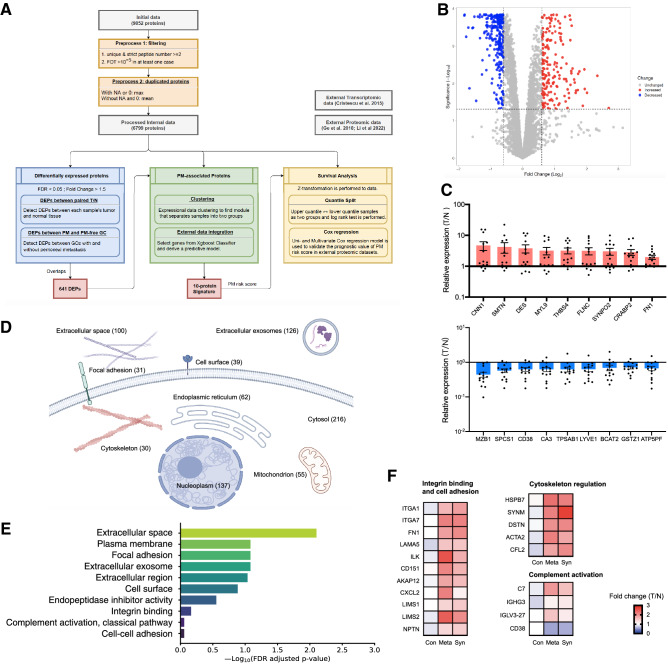
Proteomic features of GC with PM. **A** Workflow of proteomic analysis, signature screening and model construction, **B** Volcano plot of differentially expressed proteins (DEPs), **C** Top DEPs between tumor and normal that are significantly altered in GC with PM; upper panel, upregulated proteins; lower panel, downregulated proteins, **D** Subcellular distribution of DEPs annotated with Gene Ontology, **E** Top ranked pathways of DEPs, annotated with Gene Ontology and KEGG, **F** Expression of key DEPs in altered pathways in control (Con), metachronous PM (Meta) and synchronous PM (Syn) groups

As illustrated in Fig. 3A, to screen the candidate proteins associated with PM propensity in primary GC, we first selected proteins differentially expressed between tumor and normal tissues (*n* = 20), and then compared their expression between GC with PM (*n* = 14) and without PM (*n* = 6), resulting in 641 differentially expressed proteins (DEPs, Fig. 3B). In addition, we analyzed significantly altered proteins in GC with PM (*n* = 14). Upregulated proteins included CNN1, SMTN, DES and FN1, and downregulated proteins included MZB1, SPCS1, CD38 and CA3 (Fig. 3C). GO analysis of the 641 DEPs revealed that extracellular exosome and extracellular space were two most significant annotations ranked by FDR (Fig. 3D, Table S3), and other significant annotations included focal adhesion and adherens junction, suggesting that GC with PM propensity were enriched in expressional changes of tumor microenvironment components. KEGG analysis revealed that these DEPs were significantly enriched in integrin binding/focal adhesion, cytoskeleton regulation and complement activation pathways (Fig. 3E and F, Table S3).

### Identification and validation of a proteomic signature associated with PM propensity and prognosis

**Fig. 4:**
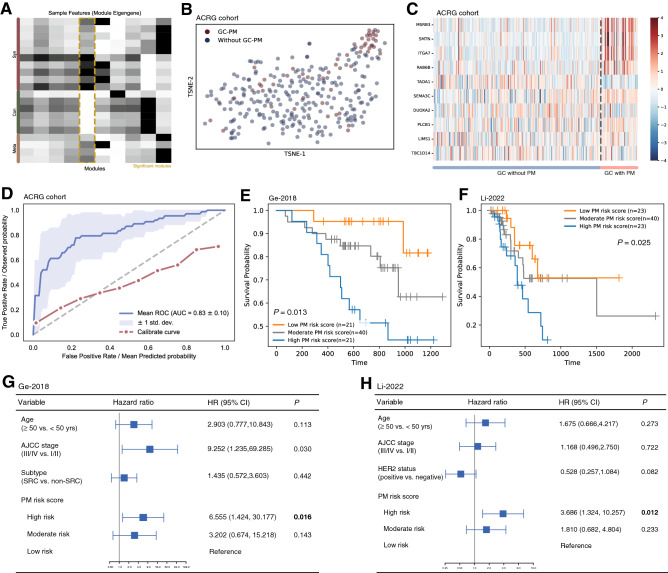
Establishment of PM-associated proteomic signature and external validation. **A** MATTE clustering of PM-associated module, **B** Heatmap depicting expression of ten proteins in GC with or without PM in ACRG cohort, **C** Dimension reduction of expression data in ACRG cohort; each dot represents one sample, **D** ROC curve of prediction model using Xgboost classifier, **E**–**F** Kaplan–Meier plot of Ge-2018 (**E)** and Li-2022 (**F**) cohort, **G**–**H** Forrest plot of multivariate Cox regression of Ge-2018 (**G**) and Li-2022 (**H**) cohort

We then attempted to obtain a protein signature associated with PM propensity in GC. First, based on our previously developed clustering method MATTE, we conducted cluster analysis that derive a PM-related module, and selected the proteins in the module with the highest signal-to-noise (Fig. 4A, *n* = 20). Subsequently, an external transcriptome-based expressional data [27] was used for further screening and verification. By performing dimension reduction of expression data, PM and PM-free samples were roughly discriminated (Fig. 4B). 10 proteins (DUOXA2, ITGA7, LIMS1, MSRB3, PLCB1, RAB6B, SEMA3C, SMTN, TADA1, TBC1D14) from the PM-related module were selected to predict whether PM occurred (Fig. 4C, the selection method described in Methods). The results showed that under the fivefold cross validation, the AUC of the average ROC curve reached 0.83 in the cohort (Fig. 4D), suggesting high accuracy of PM prediction.

The prognostic value of the 10-protein signature was further evaluated. By machine learning algorithms, a PM risk score was generated in internal cohort (Fig. S5, the weights of each protein in Tables S4). Next, we validated the model in diffuse and mixed type GC cases from two external cohorts, Ge-2018 [9] (*n* = 82) and Li-2022 [11] cohort (*n* = 86). Patients were divided as high risk (highest quartile), moderate-risk and low risk (lowest quartile) groups according to the PM risk score, and those in high-risk group had significantly shorter overall survival than low-risk group in both cohorts (*P* = 0.013 in Ge-2018 and *P* = 0.025 in Li-2022, Fig. 4E and F). Multivariate Cox regression showed that after adjusting for clinical features such as age and AJCC staging, high PM risk score still significantly correlated to poor survival (HR = 6.555, 95% CI 1.424–30.177, *P* = 0.016 in Ge-2018 and HR = 3.686, 95% CI 1.324–10.257, *P* = 0.012 in Li-2022) (Fig. 4G and H), suggesting this signature was an independent prognosticator for overall survival. Interestingly, this prognostic significance was absent in the whole Li-2022 cohort including Lauren intestinal subtype (Fig. S6, *n* = 206). These findings emphasized that the 10-protein signature was predictive of PM-associated prognosis.

### 10-protein signature is associated with TIM and treatment response

**Fig. 5:**
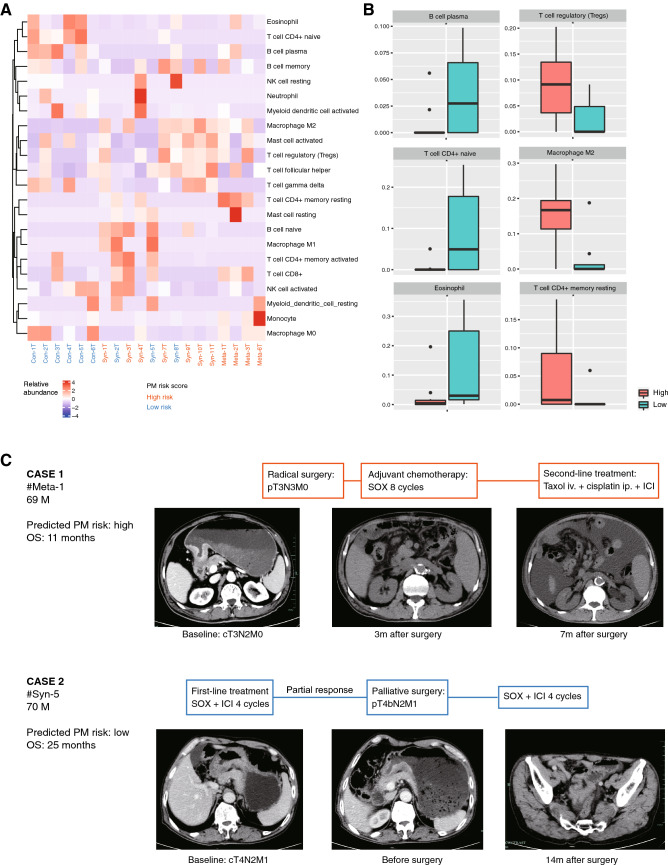
PM risk score is associated with tumor immune microenvironment and treatment response. **A** Heatmap of immune cell infiltration by CIBERSORT analysis. **B** Immune cell fractions in GC with high and low PM risk score, **C** Treatment and outcome of representative GC cases with high or low PM risk. SOX, oxaliplatin plus S-1 chemotherapy; ICI, immune checkpoint inhibitor; OS, overall survival; *P < 0.05; ns, not significant

To explore the relevance between our prognostic model and TIM, we compared the proteome-based immune cell infiltration of GC samples of high-risk group and low-risk group by CIBERSORT analysis (Fig. 5A). In 22 immune cell fractions, plasma B cell, CD4 + naïve T cell and eosinophil were significantly more abundant in low-risk group, whereas M2 macrophage, regulatory T cell and resting CD4 + memory T cell were more abundant in high-risk group (Fig. 5B). Other cell fractions such as CD8 + T cell was not significantly different between two groups. These results indicated that PM risk score was associated with TIM and might be predictive for immunotherapy response.

The clinical relevance of PM risk score was shown in representative cases (Fig. 5C). Case Meta-1, 69 years old, male, evaluated as high risk, who received radical surgery and the pathological staging was pT3N3aM0, then received 8 cycles of SOX (oxaliplatin plus S-1) adjuvant chemotherapy. 7 months after surgery, tumor relapsed as large amount of malignant ascites, and responded poorly to the second-line treatment including chemotherapy and PD-1 inhibitor. The overall survival was 11 months. Case Syn-5, 70 years old, male, evaluated as low risk, whose clinical stage was cT4N2M1 (with PM) at baseline, received first-line SOX chemotherapy plus PD-1 inhibitor. 4 cycles later, the patient developed GI bleeding, and the radiological evaluation of tumor response was partial response. Then the patient received palliative surgery, in which peritoneal metastases were observed, and the pathological staging was ypT4bN2M1. After surgery the patient received another 4 cycles of SOX plus ICI and maintained stable disease, until the tumor progressed 14 months after surgery, presenting as minor pelvic ascites. The overall survival was 25 months. These cases suggested that the PM risk score might not only be associated with aggressiveness of GC, but also with treatment response.

## Discussion

In the present study, we comprehensively confirmed genomic and proteomic heterogeneity between primary GC and PM, analyzed the distinct proteomic characteristics associated with PM progression, and then established a proteomic signature associated with the risk of PM and prognosis. This signature was also potentially associated with TIM and immunotherapy response in GC patients.

Currently, PM risk was estimated mainly according to TNM staging, especially T stage (T3-T4 were usually considered as with high risk), and GC of Lauren diffuse type was reported to have a significant tendency of developing PM [32]. However, due to the high heterogeneity within GC, clinicopathological features might not fully depict its aggressiveness and metastasis pattern. Our proteome-based prediction model would optimize the risk stratification of PM, as a tool for improving clinical management for GC patients.

In recent years, there are evolving multi-omics studies for PM of GC. Wang et al. described the genomic and immune landscape of peritoneal carcinomatosis of GC based on 43 PM cases, revealing two subtypes with discriminating response rates to chemotherapy [33]. A subsequent study used single-cell transcriptome profiling of peritoneal carcinomatosis to reveal two subtypes with different intra-tumoral heterogeneity [34]. A multi-omics analysis of malignant ascitic fluid samples and tumor cell lines from 98 patients, stratified ascites-disseminated GC into two distinct molecular subtypes: “non-EMT” subtype displaying active super enhancers (SEs), and “EMT” subtype bearing TGF-β pathway activation and high expression of TEAD-1 [35]. Unlike the above studies, our study mainly focused on proteomics of primary GC with PM, attempting to reveal key proteomic features associated with PM propensity and PM-related aggressiveness.

Consistent with previous study [8], extensive genomic heterogeneity in somatic mutations, CNVs and subclone structure was observed between primary GC and paired PM, which could be a barrier to precision medicine. The proteomic difference between paired primary GC/PM was partly due to different extracellular components such as stromal content and exosomes. Proteins enriched in PM tissues were involved in RNA binding and polysomal ribosome, suggesting more active protein synthesis in metastases.

By comparing proteomic profiles between different sample groups, we observed distinct features associated with PM progression. Enriched pathways in PM samples (compared with primary GC) included RNA binding, extracellular exosome and focal adhesion. Meanwhile, DEPs between primary GC of PM group and non-PM group were enriched in extracellular exosome, focal adhesion, cytoskeleton regulation and complement activation pathways. The overlapped pathways of the two analyses suggested key biological processes of PM. Exosomes are biologically functional extracellular vesicles comprising active factors. They can mediate metastasis of GC by promoting EMT, cancer-associated fibroblast formation, pre-metastatic niche formation and immunosuppression [36]. Focal adhesion are anchoring units and interacts between the cell-extracellular matrix and cell cytoskeleton, associated with multiple oncogenic processes such as cell proliferation, cell cycle regulation migration and chemoresistance in cancer cells [37].

The Asian Cancer Research Group (ACRG) categorizes GC into four subtypes using expression data. The MSS/EMT subtype, having the worst prognosis, was characterized by a gene expression signature correlated with EMT and a distinct tendency to develop PM [27]. It was the first large-scale transcriptional study that contains a considerably large number of subjects with PM. A large multi-omic study of early onset GC revealed that genes with significant survival differences showed stronger mRNA-protein correlations than genes with non-significant survival differences [38]. Therefore, although the data of ACRG was transcriptome-based, it is considerable to combine this dataset with our proteome-based data to screen key proteins with significant clinical implications, as these proteins were likely to have high mRNA-protein correlation. By this combination, we screened proteins associated with PM propensity, and the internal validation resulted in high accuracy.

Most proteins in our signature were involved in cancer-related mechanisms. For instance, ITGA7 was associated with cancer stemness in esophageal squamous cell carcinoma and correlates with poor prognosis in hepatocellular carcinoma [39, 40]. DUOXA2 was maturation factor of an oxidative protein DUOX2, which promotes invasion and metastasis of colorectal cancer [41, 42]. MSRB3 was reported to promote cancer by regulating genome stability and endoplasmic reticulum stress [43]. Upregulation of TADA1 was reported to promote lung squamous cell carcinoma progression [44]. RAB6B regulates intracellular membrane trafficking pathways, and its silence inhibited AKT/JNK signaling in GC [45, 46]. SEMA3C was reported to be an oncogene in multiple tumors [47]. TBC1D14 was found to inhibit lymph node metastasis by regulating autophagy in head and neck squamous cell carcinoma [48]. The biological functions of these proteins in PM progression of GC are promising aspects in future research.

Our prognostic model was derived from a PM-associated protein signature by machine learning, intending to stratify GC patients by PM-related survival. It is well established that Lauren subtype was significantly correlated to metastasis or recurrence pattern [32]. Therefore, we validated our prognostic model using two independent external proteomic datasets of diffuse/mixed GC, in which the OS could reflect PM risk to some extent. Ge-2018 was a proteomic study of 84 diffuse GC, in which 8 patients had available report of first recurrence site of peritoneal seeding. Li-2022 was a proteomic study including 206 GC patients (86 with diffuse/mixed type GC) undergoing chemotherapy or HER2 targeted therapy. Although the two cohorts had discrepant inclusion criteria, validation of our prognostic model yielded consistent results, suggesting the robustness of this model. The prognostic value of this model was absent in intestinal GC or the whole cohort, because intestinal GC is more likely to develop hematogenous metastasis, which highly affects OS. The predictive value of this model for PM-related survival in intestinal type GC still requires further cohort validation.

Tumor-infiltrating immune cells are correlated with the progression of cancer and patients’ outcome, and also affect tumor responses to immunotherapy [49]. The infiltration of macrophages M2 and Tregs was significantly negatively correlated with prognosis of colorectal cancer patients [50]. Infiltration of plasma cells as effectors of humoral immune response, were correlated with longer survival in GC [51] and breast cancer [52]. Activated CD4 + memory cells, rather than resting cells, play important role in anti-tumor immunity. Taken together, our findings revealed that GC with high PM risk tended to harbor a more immunosuppressive microenvironment, and might respond poorly to immunotherapy. Although preoperative chemotherapy could remodel TIM [53], no significant difference was observed between GC with and without preoperative chemotherapy in our cohort (Fig. S7). The two representative cases both received combined chemotherapy and immunotherapy. Meta-1 was predicted as high PM risk, and was diagnosed as locally advanced stage, but peritoneal recurrence developed shortly after radical surgery, leading to a poor survival. In contrast, Syn-5, predicted as low PM risk, although first diagnosed as advanced stage, had a much longer survival. The distinct prognosis might be partly due to their different TIM and responses to immunotherapy.

Strengths of our study included proteomic profiling of both primary GC and PM samples, machine learning and modeling integrating large-scale expressional data, and a panel of proteins associated with both PM propensity and patient outcome, which was validated at protein level in two independent cohorts. We acknowledge several limitations. First, attempting to reveal key features associated with PM, our proteomic data had a small sample size, especially that of PM samples, because the clinical scenario to acquire both primary and PM samples was rare. Second, lacking required clinical information, we have not been able to obtain external proteomic data to directly validate the predictive value of our model to PM risk or PM-free survival, but only validated its prognostic value in diffuse/mixed type GC. Although PM is the most frequent metastatic pattern in diffuse/mixed type GC, future studies of large cohorts including all Lauren subtypes of GC are warranted to validate the model.

In summary, we depicted molecular features of primary GC and PM tissues by multidimensional proteomic and genomic analysis, and characterized proteomic features that were informative for the mechanisms of PM progression. A proteomic signature was derived by machine learning algorithms to predict PM propensity and prognosis in GC. The signature was associated with TIM and potentially with immunotherapy response. Our findings could guide prophylactic intraperitoneal treatment in locally advanced GC patients with high PM risk and improve individualized disease management.

## Supplementary Information

Below is the link to the electronic supplementary material.

Supplementary file1 (XLSX 30 KB)

Supplementary file2 (PDF 2454 KB)

## Data Availability

The data in this study are available from the author for correspondence upon reasonable request.
