# Supplementary material for: Proteomic profiling of gastric cancer with peritoneal metastasis identifies a protein signature associated with immune microenvironment and patient outcome
Source: Gastric Cancer. 2023 Mar 17;26(4):504–16. doi: 10.1007/s10120-023-01379-0 (PMC10284991; doi:10.1007/s10120-023-01379-0)
Supplement: Supplementary file 2 — Supplementary file2 (PDF 2454 KB) [file 10120_2023_1379_MOESM2_ESM.pdf]

Supplementary methods

Tumor purity analysis

ABSOLUTE was performed to estimate the tumor purity as previously described[20] with the parameters: min.ploidy = 0.5, max.ploidy = 8, max.sigma.h = 0.2, copy_num_type = "total", sigma.p = 0, max.non.clonal = 1, max.neg.genome = 0.005, min.mut.af = 0.

Mutation signature analysis

Mutational signatures were characterized according to the 96-substitution classification. Based on the frequency of 96 mutation types, Nonnegative Matrix Factorization (NMF) method (v0.22) was performed to extract mutational signatures and compare them with 30 known signatures referenced in the Catalogue of Somatic Mutations in Cancer (COSMIC) database using SomaticSignatures packages (v2.24.0)[1]. The similarity of mutation signatures was evaluated with cosnine similarity > 0.9, which suggested common signatures.

HLA genotyping and neoantigen prediction

First, HLA sequences (chr6:28,477,797-33,448,354) extracted from each sample and determine HLA genotyping by HLA-HD[2]. Next, extract sequences -15 to 1000 aa from mutation site, derive potential neoantigens. Subsequently, DeepHLApan (v1.1)[3], NetMHCpan (v4.1)[4] and MHCflurry (v2.0.0)[5] was invoked to predict mutation-derived neoantigens which could bind to class I MHC molecules. By DeepHLApan, we screened peptides that are with binding score >0.5 and immunogenicity>0.5. By NetMHCpan, we selected peptides with Binding Affinity Rank <0.5% and Mass-Spectrometry Eluted Ligands Rank <0.5%. By MHCflurry we selected peptides with presentation_percentile < 2. The neoantigens from overlap of the above three algorithms are considered as neoantigens from GC samples.

Reference

1. Gehring JS, Fischer B, Lawrence M, Huber W: SomaticSignatures: inferring mutational signatures from single-nucleotide variants. Bioinformatics 2015, 31(22):3673-5.
2. Kawaguchi S, Higasa K, Shimizu M, Yamada R, Matsuda F: HLA-HD: An accurate HLA typing algorithm for next-generation sequencing data. Hum Mutat 2017, 38(7):788-97.
3. Wu J, Wang W, Zhang J, Zhou B, Zhao W, Su Z et al: DeepHLApan: A Deep Learning Approach for Neoantigen Prediction Considering Both HLA-Peptide Binding and Immunogenicity. Front Immunol 2019, 10:2559.
4. Jurtz V, Paul S, Andreatta M, Marcatili P, Peters B, Nielsen M: NetMHCpan-4.0: Improved Peptide-MHC Class I Interaction Predictions Integrating Eluted Ligand and Peptide Binding Affinity Data. J Immunol 2017, 199(9):3360-8.
5. O'Donnell TJ, Rubinsteyn A, Laserson U: MHCflurry 2.0: Improved Pan-Allele Prediction of MHC Class I-Presented Peptides by Incorporating Antigen Processing. Cell Syst 2020, 11(1):42-8 e7.

Supplementary Figures

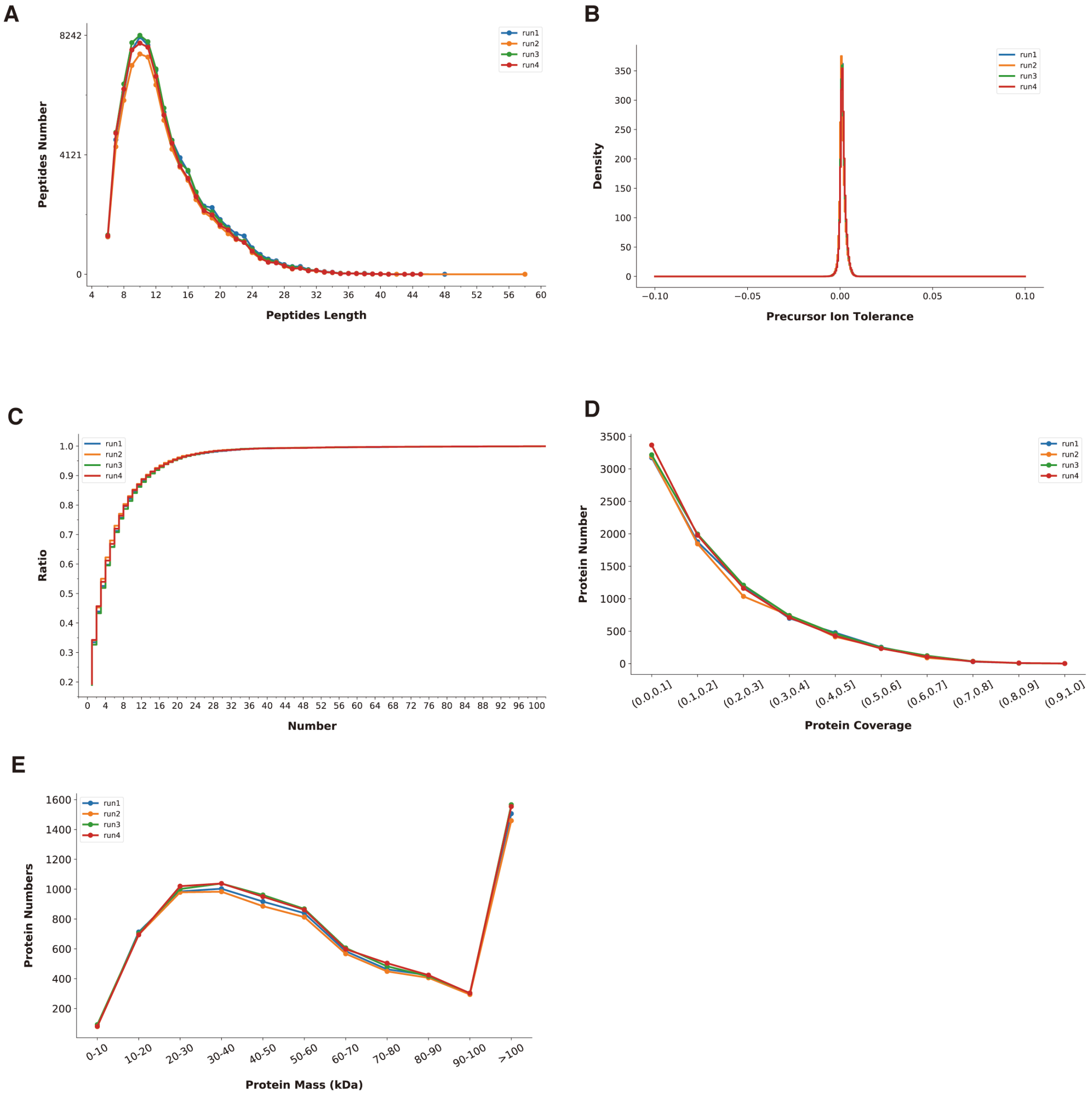

Figure S1. Quality control of proteomic data. The 40 samples (20 tumor and 20 normal tissue) were analyzed by 4 runs. (A) Distribution of peptides length; (B) Precursor ion mass tolerance; (C) Number of identified unique peptides; (D) Protein coverage of peptides. The horizontal axis represents intervals of peptide coverage length / full length of protein; (E) The distribution of protein mass.

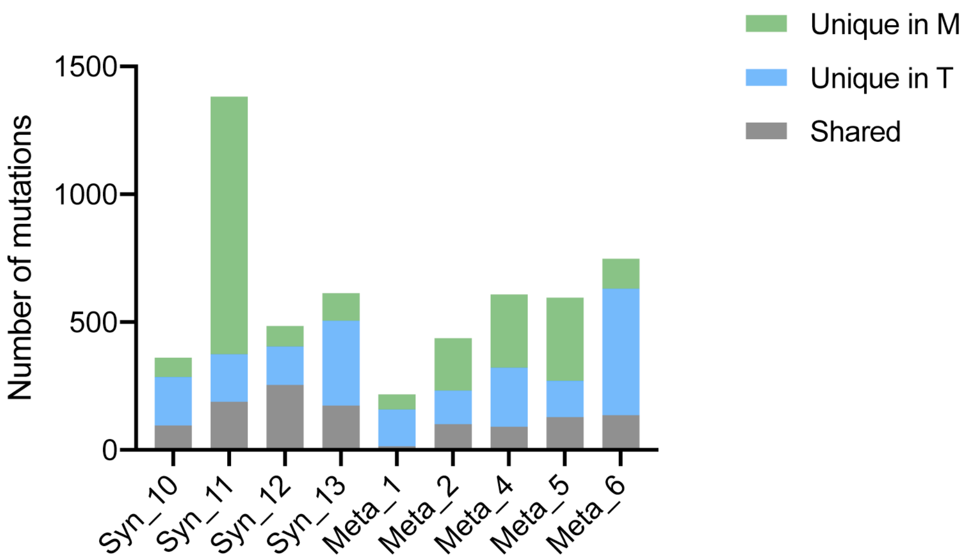

Figure S2. Somatic mutations in the primary GC (T) only, in PM (M) only, or shared between both samples.

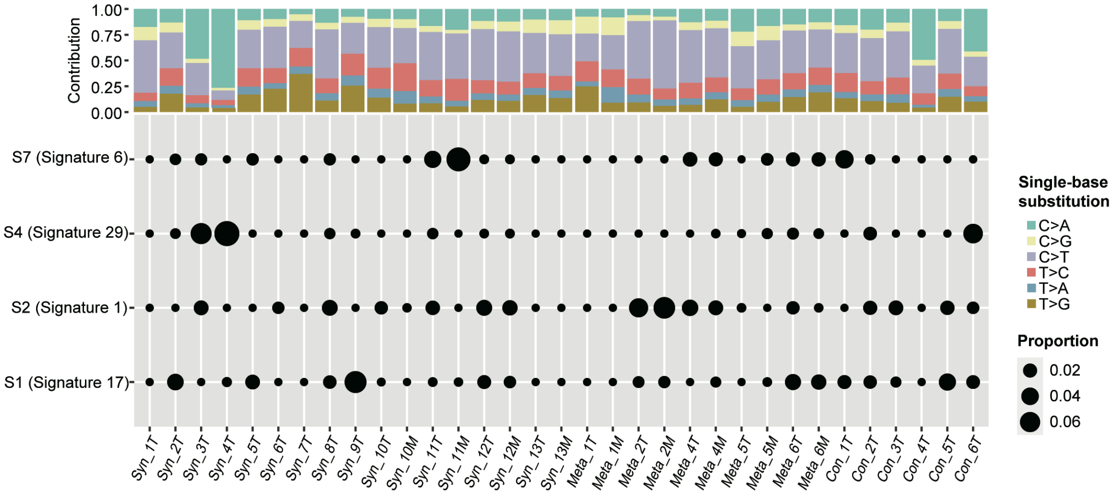

Figure S3. Mutational signature analysis of GC and PM samples.

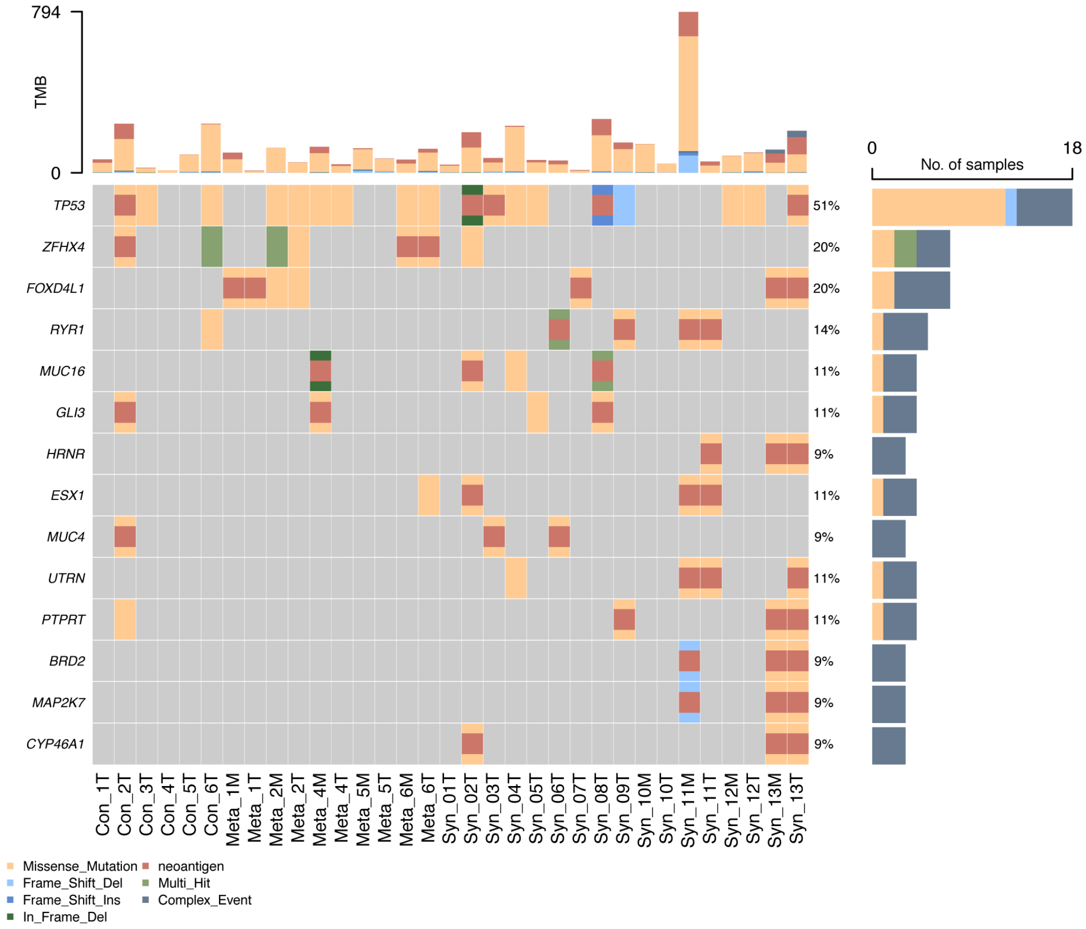

Figure S4. Neoantigen prediction of GC and PM samples. Neoantigens derived from gene mutations in at least 3 samples were depicted.

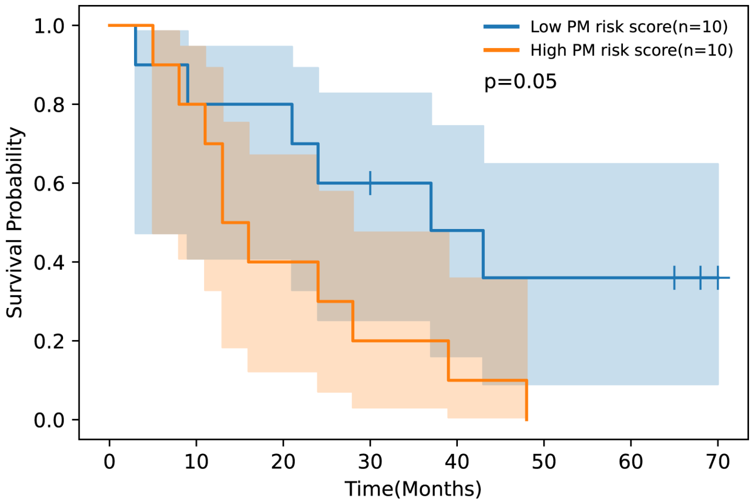

Figure S5. Prognostic value of PM risk score in internal cohort (training cohort). The PM risk was divided as high risk and low risk by median.

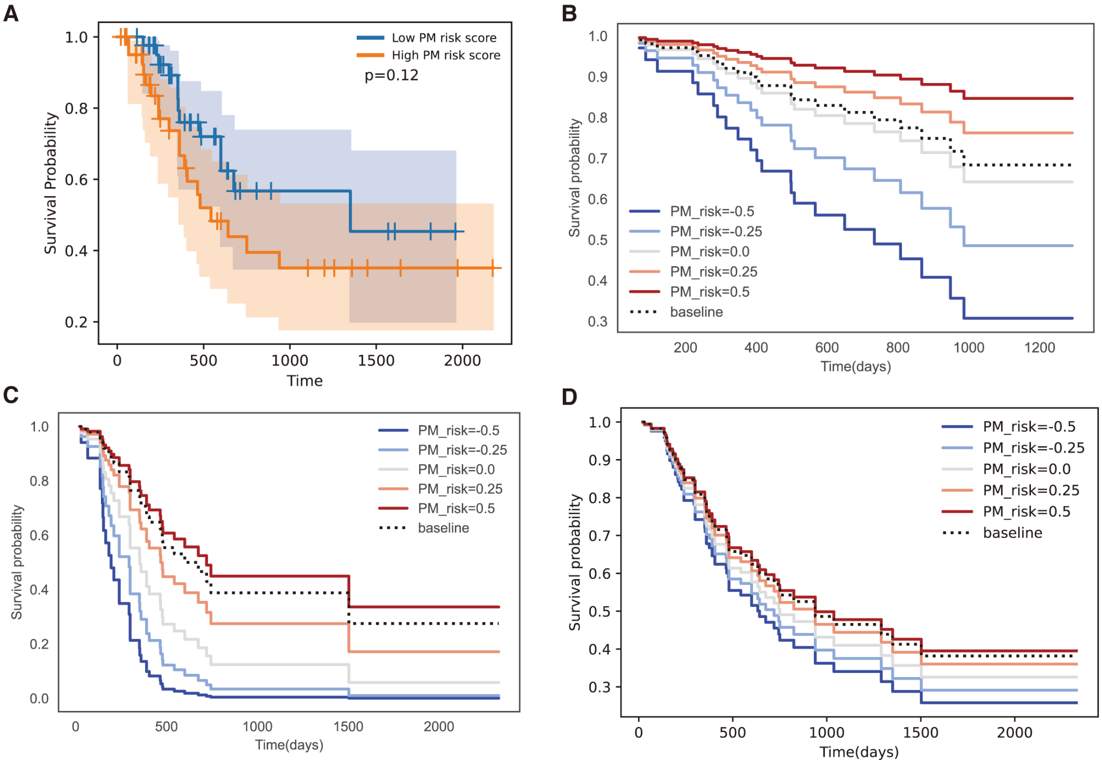

Figure S6. Prognostic value of PM risk score in validation cohorts. (A) Survival analysis of PM risk score in full cohort of Li-2022. The PM risk was divided as high risk (top 25%), low risk (bottom 25%), and moderate risk (others); (B-D) Survival analysis of PM risk score in Ge-2018 cohort (B), diffuse/mixed GC in Li-2022 cohort (C) and full cohort of Li-2022 (D). PM risk was evenly divided as 5 levels.

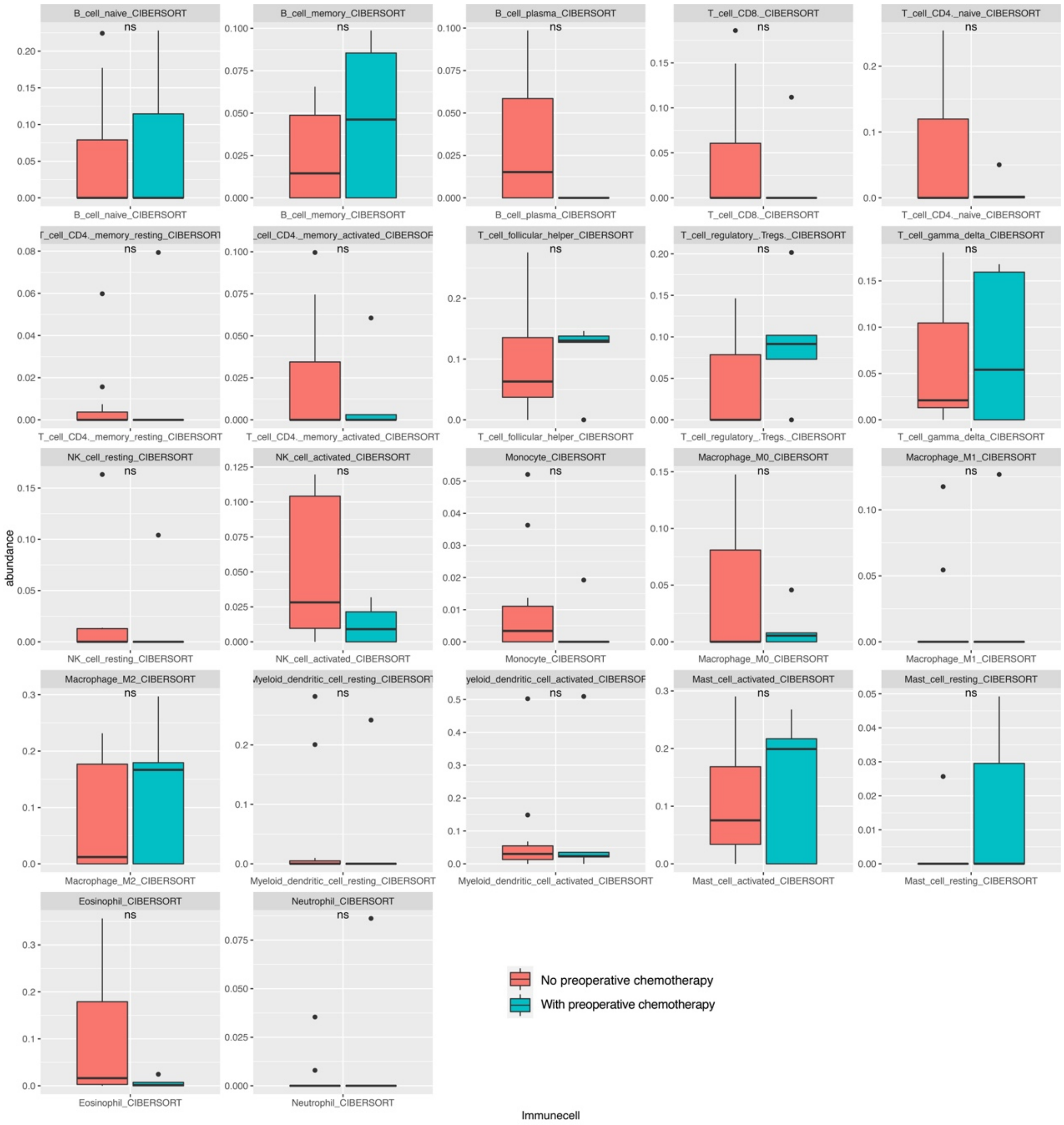

Figure S7. Immune cell fractions in GC with and without preoperative treatment. In 20 cases with proteomic data, Con-3, Syn-4, 5, 7, 9, 10 received preoperative treatment and the other 15 cases were treatment-naïve. Ns, not significant.
